# Socioeconomic Burden of Psychiatric Cancer Patients: A Narrative Review

**DOI:** 10.3390/cancers16061108

**Published:** 2024-03-09

**Authors:** Gniewko Więckiewicz, Sophie Weber, Iga Florczyk, Piotr Gorczyca

**Affiliations:** 1 Department of Psychiatry, Faculty of Medical Sciences in Zabrze, Medical University of Silesia, 40-055 Katowice, Poland; 2 First Department of Psychiatry, Institute of Psychiatry and Neurology, 02-957 Warsaw, Poland

**Keywords:** depression, psychosis, schizophrenia, addiction, anxiety, psychiatry, oncology, socioeconomic, burden, PTSD

## Abstract

**Simple Summary:**

Individuals diagnosed with cancer and experiencing mental health challenges such as depression, psychosis, anxiety, or addictions face heightened socioeconomic risks. These include increased healthcare costs, reduced treatment adherence, and a diminished quality of life. The burden of coping with both conditions simultaneously can exacerbate their impacts. This burden is also related to factors such as gender or age. Fortunately, physicians can play a crucial role in mitigating these risks. By adopting integrated care strategies, healthcare providers can address the unique needs of patients navigating the complex intersection of cancer and mental health disorders. Proactive measures, personalized support, and tailored interventions are recommended as they can significantly improve outcomes, offering hope and enhancing the overall well-being of individuals facing these dual challenges.

**Abstract:**

This comprehensive review article examines the complex overlap of affective disorders, psychoses, addictions, anxieties, post-traumatic stress disorder, and somatic symptom disorder in the context of cancer patients, and highlights the intricate interplay between psychiatric and oncological diagnoses. Based on extensive literature, it highlights the profound socioeconomic burdens that result from the coexistence of these disorders. The analysis includes the increased healthcare costs, impaired adherence to treatment, and reduced quality of life for individuals struggling with the co-occurrence of psychiatric and cancer-related problems. By synthesizing the available data through a narrative inquiry, the report aims to provide a nuanced understanding of the multiple socioeconomic challenges faced by this vulnerable patient population. The synthesis of information provides valuable insights for healthcare professionals, policymakers, and researchers alike. The aim is to promote the development of more effective and integrated care strategies tailored to the specific needs of people navigating the complicated environment of psychiatric and cancer diagnoses. Ultimately, this review should enable progress in the provision of holistic, patient-centered care for this complex intersection of health conditions.

## 1. Introduction

The co-occurrence of psychiatric disorders and cancer is a complex and multifaceted challenge that has a significant impact on patients, caregivers, and healthcare systems. Psychiatric disorders manifest as common comorbidities among cancer patients, significantly impacting their overall well-being. Addressing both the physical and psychological dimensions is imperative for optimizing the overall quality of life for cancer patients. Understanding and researching the socioeconomic burden of psychiatric illness in cancer patients is of paramount importance in the fields of oncology and mental health. This research is crucial for several reasons: First, it reveals the intricate interplay between psychological well-being and the economic challenges that individuals face in the complex landscape of cancer diagnosis and treatment. By describing the socioeconomic burden, healthcare professionals gain insight into the multiple factors that influence treatment adherence, quality of life, and overall patient outcomes. In addition, a nuanced understanding of these burdens can inform healthcare policy and resource allocation and facilitate the development of targeted interventions that address the specific needs of psychiatric cancer patients. It also serves as a basis for fostering interdisciplinary collaboration between oncologists, mental health professionals, and policymakers to develop comprehensive and patient-centered care strategies. Numerous studies have shown that psychiatric disorders contribute to a significant socioeconomic burden for both cancer patients and their caregivers [1,2,3,4,5,6,7,8]. This burden extends beyond the patients themselves and also affects their spouses and caregivers, with evidence of a higher prevalence of mental illness in the spouses of cancer patients [1]. Furthermore, the impact of psychiatric disorders is consistent across different socioeconomic status classes [9] and affects people from different social classes. The impact of psychiatric disorders on cancer treatment is reflected in the lower likelihood of oral cancer patients with mental illness undergoing surgery and adjuvant therapy [8]. In addition, pre-existing mental illness has been shown to interfere with adherence to endocrine therapy for breast cancer [10].

There is an increasing focus on the cumulative impact of psychiatric disorders in cancer patients, with depression being the most common psychiatric disorder in this population [3]. The burden of psychiatric disorders varies by cancer type and treatment, emphasizing the importance of understanding the complex interplay between physical health and psychological well-being [11,12]. Furthermore, the socioeconomic burden of psychiatric disorders remains constant regardless of differences in socioeconomic status and medication use, emphasizing the widespread nature of this problem [9].

**Figure 1:**
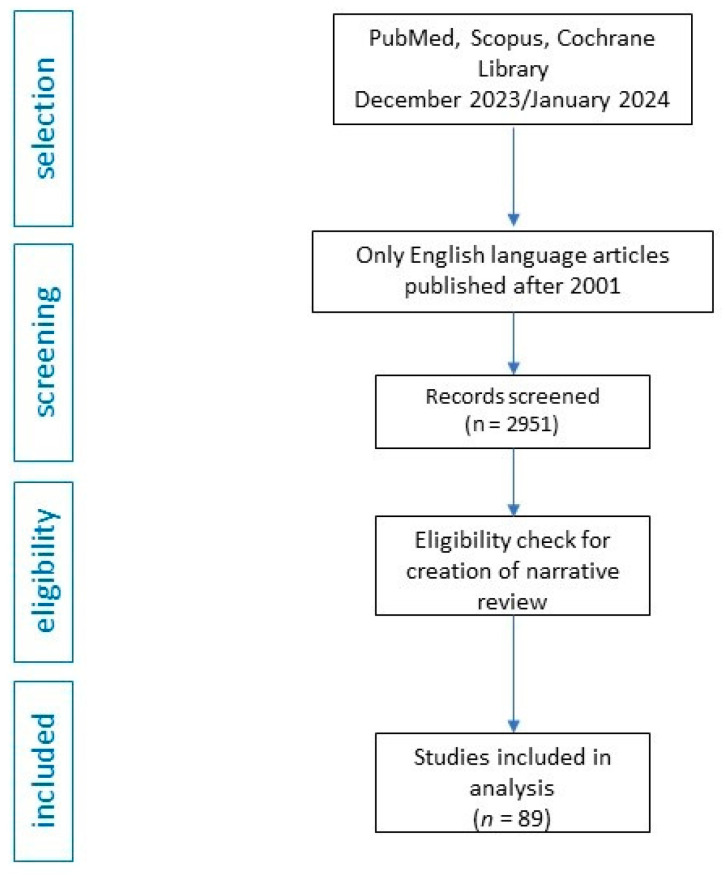
Flow chart.

The socioeconomic impact of psychiatric disorders associated with cancer is a complex and far-reaching problem that affects not only the patients themselves, but also caregivers and the healthcare system in general. The co-occurrence of psychiatric illness and cancer brings challenges in terms of access to care, adherence to treatment, and inequalities in healthcare. In order to provide comprehensive and equitable care to cancer patients with psychiatric comorbidities, it is important to understand and manage this burden. In the following article, we address the socioeconomic burden of psychiatric cancer patients. This paper examines the socioeconomic impact of people with psychiatric illness and cancer, focusing on affective disorders, psychosis, anxiety, and addictions. We also discuss the clinical, stress-related outcomes, and provide a list for physicians to consider when caring for psychiatric cancer patients. To shed light on this complicated interplay, the authors conducted a thorough search of renowned literature databases such as PubMed, Scopus, and the Cochrane Library in December 2023 and January 2024. The search used carefully selected terms that matched the focus of our work. Due to the authors’ extensive experience in the field of psychiatric cancer patients, a rigorous screening was performed, focusing on peer-reviewed articles in English published after 2001 and directly related to the central topic of this review. During the initial screening, two authors meticulously screened titles as well as abstracts and used their clinical experience to identify and select manuscripts that met the criteria for inclusion. We screened 2951 articles (title/abstract). To ensure the reliability of the information presented, we only considered data from trustworthy peer-reviewed journals. All sources that did not meet these criteria were deliberately excluded from our review. As our manuscript has the format of a narrative review, which differs from systematic reviews, we also deliberately avoided using tools such as the Preferred Reporting Items for Systematic Reviews and Meta-Analyses (PRISMA). This deliberate decision underlines our aim to provide a comprehensive synthesis of serious findings summarized in a narrative review. The flow chart is presented in Figure 1.

## 2. Socioeconomic Associations between Psychiatric Illness and Cancer

### 2.1. Affective Disorders

Patients struggling with depression and cancer often face increased healthcare costs, especially in the elderly [12]. This burden is exacerbated by factors such as unemployment, lower levels of education, and advanced stages of cancer, all of which are associated with an increased likelihood of developing depression [12]. The impact also extends to cancer survivors, as depression is a major cause of stress and severely affects their overall quality of life. From a public health perspective, the need to treat depression in cancer patients cannot be overemphasized as it plays a critical role in improving quality of life and curbing the overall burden of disease. Furthermore, the co-occurrence of depressive symptoms and cancer has been linked to increased mortality rates, highlighting the major public health challenge that this poses. In addition, caregivers of cancer patients also bear a significant burden as they struggle with emotional problems and reduced quality of life, underscoring the far-reaching impact of this dual diagnosis [13]. The responsibility of caring for cancer patients is associated with symptoms of anxiety, depression, and impaired quality of life, highlighting the cascading effect of this comorbidity on the wider support network of cancer patients [3,13]. Furthermore, the socioeconomic impact of cancer and depression is evident in the area of healthcare expenditure, as people with depression incur higher total, outpatient, and prescription costs compared to patients without depression [12].

Individuals struggling with bipolar disorder carry a significant burden of general medical comorbidity, which contributes significantly to the overarching socioeconomic impact [14]. Furthermore, the estimated lifetime prevalence of bipolar disorder in Europe is 3.9%, highlighting the large number of people affected by this condition [15]. Caregivers supporting individuals with bipolar disorder face burdens that impact health, mental well-being, and financial situations, emphasizing the far-reaching impact on the support network of these patients [16]. In addition, the families of people with bipolar disorder also face significant burdens, further emphasizing the far-reaching impact of this condition [17]. From a public health perspective, it is imperative to address the burdens faced by caregivers and families of people with bipolar disorder in order to alleviate the overall societal impact. Furthermore, the burden on informal caregivers may fluctuate over time due to the episodic and cyclical nature of the disorder, indicating enduring effects for caregivers [18]. The impact of bipolar disorder extends beyond immediate health concerns and affects overall functioning as well as socioeconomic status, affecting both patients and their close relatives. The complicated interaction between bipolar disorder treatment and cancer treatment can lead to increased healthcare costs, reduced ability to work, and strained social relationships, increasing the overall socioeconomic burden. In addition, the impact of a family predisposition to psychiatric disorders on overall functioning underscores the need for robust support systems for individuals struggling with the co-occurring challenges of bipolar disorder and cancer, as well as for their families. Public health interventions must recognize the multiple challenges posed by this comorbidity and provide tailored support to improve patient outcomes and effectively alleviate the socioeconomic burden [19]. In addition, individuals with bipolar disorder have been associated with an increased risk of cancer, adding to the socioeconomic burden and public health impact [20]. The socioeconomic burden of individuals with both bipolar disorder and cancer is a major public health concern. Addressing this problem requires comprehensive interventions aimed at mitigating the impact and ultimately improving patient outcomes.

### 2.2. Psychotic Disorders (e.g., Schizophrenia)

Schizophrenia, psychotic disorders, and cancer together represent a significant socioeconomic burden that impacts public health and drives up treatment costs. Particularly in developing countries, people with schizophrenia face economic barriers associated with the cost of antipsychotic treatment [21]. Caregivers caring for people with schizophrenia speak of an increased burden that includes impacts on family functioning, psychological challenges, financial burdens, and health-related concerns [22]. The burden associated with schizophrenia is exacerbated by a patient’s limited ability to work and contributes to the economic burden on a family [23]. In addition, the COVID-19 pandemic is expected to disproportionately affect people with schizophrenia and related disorders, compounding their problems [13].

Conversely, cancer represents a significant socioeconomic burden, with both direct and indirect costs rising steadily over time [24]. The burden of cancer is influenced by socioeconomic status, as evidenced by a notable burden of smoking that is prevalent in lower socioeconomic groups [25]. In addition, people with schizophrenia are less likely to attend cancer screenings, resulting in advanced cancer being detected at initial presentations, which in turn can lead to higher treatment costs [26].

When talking about the socioeconomic burden of this particular patient group, it is worth mentioning the stigma associated with schizophrenia, which often leads to delays in seeking and receiving a diagnosis, which in turn affects the initiation of treatment. The stigma and discrimination faced by people with schizophrenia are identified as common barriers to accessing treatment and contribute to delays in seeking mental health care [27]. This delay due to stigma can lead to potential complications such as suicide, violence, harm to others, and a deterioration in the ability to manage one’s own physical health [28]. In addition, the negative effects of stigma are particularly evident in the delay in seeking treatment [29]. Stigma also poses a challenge to interpersonal and professional relationships, further prolonging the time to diagnosis and the initiation of treatment for schizophrenia [30].

Internalized stigma, resulting from the internalization of societal stigma by individuals with schizophrenia, emerges as a notable factor contributing to delays in seeking treatment [31]. In addition, self-stigma, which is influenced by family dynamics, is associated with reduced quality of life and can cause significant distress to patients with schizophrenia, which in turn contributes to delays in seeking diagnosis and treatment [32]. Mitigating the effects of stigma is of paramount importance for mental health policy, particularly in schizophrenia, as it significantly affects the timely diagnosis and treatment of the condition [33]. This highlights the urgent need for targeted interventions to address and reduce stigma in mental health care, which appears to be particularly important for psychotic patients with comorbid cancer—for cancer patients without comorbidities, stigma is a significant problem as it can have a detrimental impact on their psychological well-being and quality of life. Research shows that cancer patients, particularly those diagnosed with lung cancer, often face stigma from various sources, including friends, family, and healthcare providers [34,35,36]. The stigmatization of cancer patients correlates with negative psychological outcomes, such as depression, anxiety, and psychological health problems [37,38,39]. Furthermore, stigma can affect social support for cancer patients and their caregivers, leading to additional psychosocial challenges [40]. It is evident that cancer stigma is a complex issue that requires comprehensive understanding and targeted interventions to mitigate its negative impact on patients’ well-being, and that it can undoubtedly have an increased negative impact on the group of psychiatric cancer patients, especially patients with psychotic disorders, as they are objectively among the most vulnerable groups of psychiatric patients.

### 2.3. Addictions

The socioeconomic challenges faced by people with addiction problems are complex and have significant consequences both for those affected and for society as a whole. Addiction, whether related to substances such as drugs and alcohol, activities such as gambling, or behaviors such as Internet or smartphone use, is considered a major public health problem with significant socioeconomic implications [41,42,43,44,45,46,47]. The impact of addiction on an individual’s socioeconomic status manifests itself in various facets and includes economic hardship, homelessness, and incarceration [42,44,48]. In addition, addiction correlates with a spectrum of mental health problems, such as depression, anxiety, and low self-esteem, which can exacerbate the socioeconomic challenges faced by those affected [49,50].

The socioeconomic burden of addiction goes beyond the individual impact and extends to broader societal structures. For example, addiction causes significant damage to the health and socioeconomic positions of those affected, leading to additional burdens on society [43,47]. Furthermore, addiction is associated with increased healthcare costs, increased hospitalization, and unfavorable clinical outcomes, contributing to the overall socioeconomic impact [51]. In addition, marginalized groups, including people with mental health or addiction problems, those with a low socioeconomic status, or those living in rural and remote areas, may face barriers to accessing essential health services, including clinical trials, exacerbating existing socioeconomic inequalities [52].

The relationship between addiction and socioeconomic status is complicated and influenced by various factors, including cultural and macroeconomic conditions [53,54]. Studies have emphasized the relationship between addiction and factors such as poverty, a low socioeconomic status, and disadvantaged living conditions, highlighting the need to address these socioeconomic determinants in addiction prevention and treatment initiatives [55]. Furthermore, addiction has been identified as a major public health problem with a widespread prevalence across all socioeconomic groups, emphasizing the need for comprehensive strategies to address the socioeconomic impact of addiction [45]. 

The socioeconomic challenges faced by people struggling with both addiction and cancer are more profound. The confluence of addiction and cancer poses particular challenges, especially for adolescents and young adults with cancer, who are particularly vulnerable to financial hardship [56]. Adolescents and young adults with cancer may face inadequate insurance coverage, limited financial resources, and significant interruptions in employment, resulting in increased financial strain during and after treatment [57]. In addition, the financial burden of cancer patients can hinder social development and impact education, employment, and financial self-sufficiency, particularly in young adults [58].

Furthermore, the financial burden associated with cancer can be exacerbated by the presence of addiction problems. People with addiction may already face disadvantages in employment, lower educational attainment, and poorer mental health, exacerbating their overall socioeconomic challenges [59]. The co-occurrence of addiction and cancer can further exacerbate the financial burden, leading to hardship and reduced quality of life for cancer survivors [60].

As far as dependence is concerned, it should be remembered that opioids are often used to treat pain in oncological diseases. Opioid dependence in cancer patients is a complex and multifaceted challenge that requires a thorough understanding of the contributing factors and the formulation of effective treatment strategies. The use of opioids in the treatment of cancer pain remains critical to relieving pain and improving patients’ quality of life; however, the potential for opioid dependence in this population emphasizes the need for a cautious and balanced approach to opioid prescribing and monitoring. The socioeconomic issues faced by individuals struggling with both addiction and cancer are significant and intertwined, and include financial stress, barriers to employment, and decreased quality of life. Interventions are needed that address the unique intersection of addiction and cancer, particularly for vulnerable populations such as young people.

### 2.4. Anxiety Disorders

The co-occurrence of cancer and anxiety disorders creates a complex web of socioeconomic challenges that significantly impact the well-being and healthcare of those affected. Studies have highlighted the prevalence of anxiety disorders in cancer patients, with variables such as gender, age, and cancer type contributing to an increased risk of anxiety and depression [61,62]. The co-occurrence of anxiety disorders and cancer can increase the financial burden on individuals, particularly due to the additional costs of mental health care and the impact of anxiety on employment and productivity [61,63].

In addition, anxiety disorders in cancer patients have been associated with a higher likelihood of perceiving cancer communication as ambiguous, suggesting potential difficulties in understanding and coping with cancer diagnosis and treatment. This may further impact an individual’s ability to navigate the healthcare system and make informed decisions [64]. In addition, stress caused by the COVID-19 pandemic has been identified as a factor negatively impacting cancer patients’ anxiety levels, exacerbating the impact of a cancer diagnosis and leading to a significant increase in traumatic symptoms [65].

The socioeconomic consequences of anxiety disorders associated with cancer extend beyond the diagnosed patient and also affect their caregivers. Research suggests that the caregivers of cancer patients may also experience increased health anxiety, particularly when faced with cancer experiences on behalf of a patient. This can lead to increased psychological stress and potential impacts on their quality of life [66].

In addition, the prevalence of anxiety disorders tends to be higher in women, and individuals with a lower socioeconomic status are more vulnerable to stressful life events, which increases the risk of common mental disorders such as anxiety and depression. This highlights the overlap between socioeconomic differences and mental illness in relation to cancer [67,68].

The co-occurrence of anxiety disorders and cancer presents a multifaceted challenge with significant socioeconomic implications, including an increased financial burden, difficulties in communication and decision making related to cancer, and increased psychological distress for both cancer patients and their caregivers. These complicated socioeconomic issues require comprehensive support systems and interventions that take into account the unique intersection of anxiety disorders and cancer, particularly considering factors such as gender, age, and socioeconomic inequalities.

### 2.5. Post-Traumatic Stress Disorder (PTSD)

The socioeconomic challenges faced by oncology patients with post-traumatic stress disorder (PTSD) represent a multifaceted problem with profound implications for both patients and healthcare systems. Several factors contribute to this burden: financial difficulties, reduced ability to work, and increased healthcare utilization.

Research by Smith et al. has identified socioeconomic predictors associated with increased financial burdens of cancer treatment, including factors such as a lack of health insurance, lower income, unemployment, and a younger age at cancer diagnosis. These factors may exacerbate the financial difficulties of oncology patients with PTSD, resulting in an increased economic burden and limited access to essential care [69].

In addition, the co-occurrence of PTSD and traumatic brain injury (TBI) has been associated with fewer treatment options and increased prognostic factors for chronicity, resulting in significant disability and socioeconomic burden [70]. In addition, specific sociodemographic and socioeconomic characteristics, illness-related stress, family relationships, strain characteristics, coping styles, and support have been identified as relevant risk/protective factors for PTSD in caregivers of adult patients with serious medical illness [71]. This underscores the notion that the socioeconomic context of caregivers may impact the burden of PTSD in the field of oncology care.

In addition, PTSD is associated with a significant economic burden on society, with estimates suggesting a substantial financial impact [72]. This economic burden compounds the overall socioeconomic challenges faced by oncology patients with PTSD, affecting their quality of life and access to resources.

The impact of PTSD on work-related impairments and reduced ability to work is also well documented, highlighting the wide-ranging socioeconomic consequences of PTSD in oncology patients [73]. This impaired performance can lead to financial stress and reduced productivity, which in turn contributes to the overall socioeconomic burden.

### 2.6. Somatic Symptom Disorder

The socioeconomic complexity faced by people with somatic symptoms and cancer significantly affects their general well-being and quality of life, and presents them with multiple challenges. These include financial burdens, psychological problems, and the impact of traumatic events on mental health. Smith et al. found that financial problems are common among cancer patients, particularly among younger and socioeconomically disadvantaged individuals. These financial difficulties correlate with poorer adherence to treatment and reduced health-related quality of life, highlighting the significant socioeconomic impact on this patient group [74]. In addition, Swainston et al. pointed out that worrying about COVID-19 in women who have breast cancer can increase the risk of developing affective disorders such as anxiety and depression. The psychological toll of the COVID-19 pandemic places an additional distress and may exacerbate socioeconomic challenges for people with cancer and somatic symptoms [75]. Nipp et al. pointed out the considerable proportion of cancer patients who exhibit symptoms of post-traumatic stress disorder (PTSD) in hospitals, which are associated with an increased physical and psychological symptom burden. This suggests that the psychological after-effects of cancer and its treatment contribute to the socioeconomic burden of these patients [76]. In addition, Mukherjee et al. emphasized the prevalence of psychiatric diagnoses such as depressive disorders, adjustment disorders, and anxiety in breast cancer patients accessing psycho-oncology services. These psychiatric conditions exacerbate the socioeconomic challenges faced by people struggling with cancer and somatic symptoms [77]. This highlights the wider socioeconomic impact of cancer care, through to the crucial role that nurses play in supporting patients with somatic symptoms and cancer.

## 3. Discussion

Living with a psychiatric illness can present a variety of challenges that go beyond the symptoms of the illness itself. Mental illness affects not only an individual’s cognitive and emotional well-being, but also their daily functioning, relationships, and overall quality of life. It is important to recognize that the impact of psychiatric illness is highly individualized, as the severity, duration, and specific symptoms can vary greatly across disorders. There are a few things that come to mind when thinking about important, stressful things to consider when caring for a psychiatric patient: A pervasive problem faced by people with psychiatric disorders is the stigma associated with mental illness. Despite increasing awareness, many people still have misconceptions and stereotypes about people with psychiatric illness. This stigmatization can lead to discrimination, prejudice, and social isolation, which exacerbate the challenges those affected face in managing their illness. Fear of judgment can prevent sufferers from seeking help, disclosing their diagnosis, or participating fully in social activities. Maintaining employment can be a major hurdle for people with psychiatric disorders. Symptoms such as mood swings, anxiety, or poor concentration can affect work performance. In addition, the stigma surrounding mental health can affect career advancement opportunities and job retention. In some cases, those affected are discriminated against during the hiring process or encounter a work environment that is not understanding or accommodating of mental illness. The symptoms of illnesses such as depression and anxiety can make it difficult for those affected to participate in social activities, maintain friendships, or attend family gatherings. This isolation can exacerbate feelings of loneliness and contribute to a cycle of deteriorating mental health. Maintaining healthy relationships can be challenging for people with psychiatric illness. The impact of symptoms such as mood swings, irritability, and behavioral changes can strain interpersonal relationships. Family members may have difficulty understanding or coping with the challenges of psychiatric illness, leading to conflict and strained relationships. Effective communication and a supportive network are crucial to overcoming these difficulties. Access to mental health care varies around the world, and, even in regions with established healthcare systems, access to appropriate and timely treatment can be a problem. Long waiting lists, the limited availability of mental health professionals, and financial barriers can prevent people from receiving the treatment they need. This lack of access can lead to delayed diagnosis and treatment, which in turn leads to more severe symptoms and greater difficulty in managing the condition. Medication is an important part of treatment for many psychiatric disorders; however, these medications often have side effects that can impact a person’s physical health, daily functioning, and overall well-being. Weight gain, lethargy, sexual dysfunction, and cognitive impairment are some common side effects that sufferers struggle with, further complicating their daily lives. People with psychiatric disorders are at an increased risk of developing co-occurring substance use disorders. Some turn to substances to cope with the distressing symptoms of their mental illness. Substance abuse can complicate treatment and recovery, leading to a vicious cycle of worsening mental health and increased dependence on substances. Adherence to treatment plans can be challenging for people with psychiatric illness. Factors such as forgetfulness, a lack of understanding of the severity of the illness, or concerns about the side effects of medication can contribute to treatment non-adherence. Poor adherence to treatment can hinder recovery and make it difficult for sufferers to manage their symptoms effectively.

When a person with a pre-existing mental health disorder is diagnosed with cancer, the intersection of physical and mental health presents a complex and difficult situation, and can be considered much harder to navigate for a physician. Both cancer and mental health disorders can have a significant impact on a person’s life. Below are some of the aspects that can complicate the lives of people with mental health disorders who have been diagnosed with cancer: The emotional toll of a cancer diagnosis is profound and can exacerbate existing mental health problems. Feelings of fear, anxiety, and uncertainty about the future can intensify, leading to increased depressive symptoms or a worsening of other mental health conditions. Coping with the emotional burden of a cancer diagnosis and a pre-existing mental health disorder can be overwhelming. The process of making treatment decisions becomes even more complicated when a person with a mental health disorder is diagnosed with cancer. Mental illness can affect a person’s ability to process information, make decisions, and adhere to treatment plans. Individuals may struggle with feelings of hopelessness, fear of the side effects of treatment, or difficulty understanding complex medical information, which can impact their ability to actively participate in decisions about their cancer treatment. People with mental disorders often take psychotropic drugs, and the introduction of cancer treatments can lead to potential interactions between cancer drugs and psychotropic drugs. Medical teams must carefully consider these interactions to ensure that the cancer treatment is effective without jeopardizing the patient’s mental stability. Coping with a cancer diagnosis and undergoing treatment can be very stressful. For people with mental health disorders, the added stress can contribute to a worsening of their psychiatric symptoms. Stress has been shown to impact immune function and overall well-being, and can affect an individual’s ability to cope with cancer treatments and manage both conditions simultaneously. Having a strong support system is crucial during cancer; however, it can already be difficult for people with mental health disorders to maintain social connections due to stigmatization or social withdrawal. The added stress of a cancer diagnosis can further strain relationships or lead to increased isolation, impacting an individual’s ability to receive emotional support at a critical time. People with mental health disorders often rely on certain coping mechanisms to manage their symptoms. A cancer diagnosis can disrupt these tried and tested coping strategies, leading to increased stress and a potential deterioration in mental health. It is crucial to work with healthcare professionals to develop adaptive coping mechanisms that can deal with both a cancer diagnosis and mental health challenges. Accessing cancer treatment and adhering to a rigorous treatment plan can be challenging for anyone, but it may be especially difficult for people with mental health disorders. Factors such as transportation, financial constraints, and the need for additional psychosocial support during cancer treatment can impact a person’s ability to consistently engage in cancer treatment. The focus on cancer treatment can take a back seat to ongoing mental health treatment. The mental health treatment plan may need to be adjusted to accommodate the additional stressors and challenges that cancer brings. This requires coordination between oncology and mental health professionals to ensure comprehensive and integrated care. Once cancer treatment is complete, patients may face particular issues. Fear of cancer recurrence, altered body image, and the long-term physical as well as psychological effects of cancer treatment can lead to ongoing mental health problems. Coping with these issues requires a holistic approach that takes into account both the physical and psychological well-being of an individual. For people facing advanced-stage cancer, end-of-life planning is a crucial aspect of their journey. This process can be emotionally distressing, causing existential worry, anxiety, and depression. People with mental health conditions may need additional support to manage these discussions and decisions and ensure that their wishes and emotional well-being are taken into account.

It is worth noting that untreated mental health symptoms can significantly impact the medical trajectories of oncological patients. Psycho-oncological distress not only affects the quality of life of patients but also influences their compliance with treatment and follow-up schedules [78]. This can lead to suboptimal drug therapy, patient counseling, and symptom management, which are crucial aspects of oncological care [79]. Moreover, the emotional labor experienced by medical oncologists in providing care to oncological patients can be substantial, especially in the absence of adequate mentoring and feedback loops [80]. Additionally, it is worth noting that patients with common mental disorders have a generally considerably higher cancer mortality risk than the general population [81].

Furthermore, the impact of untreated mental health symptoms extends to medical education, as it is crucial for healthcare professionals, including primary care physicians and nurses, to be well-educated on the psychological aspects of cancer care to ensure that patients receive comprehensive support [82,83]. The role of pharmacists in oncology pharmacy services is also vital, as they play a significant part in supportive care management and directly impact patient outcomes [84,85]. Additionally, the shift of oncology inpatient care to outpatient care has led to a decreased number of oncology admissions to hospitals, posing challenges in retaining expert oncology nurses and emphasizing the need for critical care training for oncology nurses preparing to care for high-acuity oncology patients [86,87].

The fact that many cancer patients do not have access to mental health services is a notable gap in care, as they rely heavily on medical providers who may not have the confidence or expertise to prescribe necessary psychotropic drugs or guide patients to mental health services. Often, oncological patients meet emotional struggles when diagnosed [88]. This lack of mental health support in the context of cancer treatment can result in patients’ psychological issues not being addressed. Medical providers, who may be uncomfortable prescribing psychotropic drugs or unfamiliar with referring patients to mental health services, face a dilemma when it comes to adequately addressing the mental well-being of people undergoing cancer treatment. Recognizing and closing this gap are critical to providing holistic and comprehensive care for cancer patients and highlight the need for greater integration of mental health services into the broader spectrum of cancer care. Individuals experiencing elevated anxiety levels were inclined to postpone seeking medical attention, which encompassed cancer screenings and diagnostic assessments. Such delays in seeking care may contribute to the advancement of cancer to more advanced stages, diminishing the prospects of successful treatment and survival. Consequently, anxiety has the potential to instigate the avoidance of essential medical care, including consultations with oncologists [89].

To summarize, physicians—especially psychiatrists and oncologists—should keep twelve very important points in mind when caring for psychiatric cancer patients. These points might reduce the burden on patients and are as shown in Table 1.

By potentially reducing the burden, physicians could potentially significantly improve patients’ quality of life.

Limitations of the present narrative review must be acknowledged to provide a comprehensive understanding of the scope and context of the socioeconomic burden among psychiatric cancer patients. Firstly, the inclusion of studies may have been subject to publication bias, as research with positive findings may be more likely to be published. Additionally, the review’s scope is inherently limited by the available literature up to the knowledge cutoff date in January 2024, and there may be relevant studies published after this date. Furthermore, variations in study methodologies, sample sizes, and demographic characteristics among the included studies may introduce heterogeneity, limiting the ability to draw direct comparisons or establish causal relationships. The complexity of socioeconomic factors, including regional disparities, cultural nuances, and evolving healthcare policies, may not have been fully captured, potentially influencing the generalizability of findings. Moreover, the multifaceted nature of psychiatric disorders and cancer adds an extra layer of complexity, making it challenging to disentangle the specific socioeconomic contributors to the overall burden. No tools to assess the quality and heterogeneity of the studies were used. Despite these limitations, this narrative review serves as a valuable synthesis of existing knowledge and a foundation for future research endeavors aiming to comprehensively explore the intricate interplay between psychiatric conditions, cancer, and socioeconomic challenges.

## 4. Conclusions

The simultaneous presence of cancer and psychiatric disorders, with a notable focus on affective disorders, schizophrenia, addiction, and anxiety, substantially amplifies the socioeconomic strain on individuals. This impact extends to their financial stability, healthcare decision making, and overall quality of life. Successfully tackling these intricate socioeconomic challenges necessitates holistic interventions that recognize the unique intersection of cancer and psychiatric illness, particularly within the context of socioeconomic disparities. These factors should be integral to the formulation of health policy measures, underscoring the importance of further research in this domain, with a strong encouragement for systematic reviews focusing on specific diseases.

## Figures and Tables

**Table 1:** Factors that might reduce burdens and explanations.

Factor	Explanation
Individualized approach	Tailor treatment plans to the specific combination of mental disorder and cancer, taking into account individual needs, preferences, and values.
Collaborative care	Facilitate communication between oncology and mental health professionals to ensure comprehensive and coordinated support.
Clear and empathetic communication	Ensure a sensitive communication style and that information is conveyed clearly and sensitively to reduce anxiety.
Mental health stability assessment	Assess the stability of a patient’s mental health before starting cancer treatment, working with mental health professionals.
Regular mental health check-ups	Conduct routine mental health check-ups during medical appointments to address concerns immediately and provide ongoing sup-port.
Support coping mechanisms	Identify and encourage the integration of adaptive coping mechanisms for managing mental health throughout cancer treatment.
Informed consent and shared decision making	Facilitate informed decision making by discussing the potential impact of cancer treatment on mental health and involving those affected in shared decision making.
Managing stigma and fears	Acknowledge and address the stigma associated with mental health and cancer by encouraging open discussions and providing accurate information.
Family and social support	Recognize the importance of a support network and involve family and friends to actively participate in emotional care and support.
Survivorship planning	Discuss survivorship planning issues early, including concerns about cancer recurrence and long-term side effects, and emphasize ongoing mental health support.
Planning for end-of-life and palliative care	Approach end-of-life conversations sensitively and work with palliative care teams to address emotional needs in advanced stages of cancer.
Accessible mental health resources	Ensure access to mental health resources and provide information about support groups, counseling services, and crisis intervention throughout the cancer journey.

## Data Availability

Data are contained within the article.
